# Online solid phase extraction liquid chromatography tandem mass spectrometry (SPE-LC-MS/MS) method for the determination of sucralose in reclaimed and drinking waters and its photo degradation in natural waters from South Florida

**DOI:** 10.1186/1752-153X-7-141

**Published:** 2013-08-22

**Authors:** Sudha Rani Batchu, Natalia Quinete, Venkata R Panditi, Piero R Gardinali

**Affiliations:** 1 Department of Chemistry and Biochemistry, Florida International University, 3000 NE 151st ST, FIU Biscayne Bay Campus, MSB-356, North Miami, FL 33181, USA; 2 Southeast Environmental Research Center (SERC), Florida International University, Miami, FL, USA

**Keywords:** Sucralose, Artificial sweetener, Online SPE, Reclaimed waters, Drinking water, Photo degradation, Degradation products, High resolution mass spectrometry

## Abstract

**Background:**

Sucralose has gained popularity as a low calorie artificial sweetener worldwide. Due to its high stability and persistence, sucralose has shown widespread occurrence in environmental waters, at concentrations that could reach up to several μg/L. Previous studies have used time consuming sample preparation methods (offline solid phase extraction/derivatization) or methods with rather high detection limits (direct injection) for sucralose analysis. This study described a faster and sensitive analytical method for the determination of sucralose in environmental samples.

**Results:**

An online SPE-LC–MS/MS method was developed, being capable to quantify sucralose in 12 minutes using only 10 mL of sample, with method detection limits (MDLs) of 4.5 ng/L, 8.5 ng/L and 45 ng/L for deionized water, drinking and reclaimed waters (1:10 diluted with deionized water), respectively. Sucralose was detected in 82% of the reclaimed water samples at concentrations reaching up to 18 μg/L. The monthly average for a period of one year was 9.1 ± 2.9 μg/L. The calculated mass loads per capita of sucralose discharged through WWTP effluents based on the concentrations detected in wastewaters in the U. S. is 5.0 mg/day/person. As expected, the concentrations observed in drinking water were much lower but still relevant reaching as high as 465 ng/L. In order to evaluate the stability of sucralose, photodegradation experiments were performed in natural waters. Significant photodegradation of sucralose was observed only in freshwater at 254 nm. Minimal degradation (<20%) was observed for all matrices under more natural conditions (350 nm or solar simulator). The only photolysis product of sucralose identified by high resolution mass spectrometry was a de-chlorinated molecule at m/z 362.0535, with molecular formula C_12_H_20_Cl_2_O_8_.

**Conclusions:**

Online SPE LC-APCI/MS/MS developed in the study was applied to more than 100 environmental samples. Sucralose was frequently detected (>80%) indicating that the conventional treatment process employed in the sewage treatment plants is not efficient for its removal. Detection of sucralose in drinking waters suggests potential contamination of surface and ground waters sources with anthropogenic wastewater streams. Its high resistance to photodegradation, minimal sorption and high solubility indicate that sucralose could be a good tracer of anthropogenic wastewater intrusion into the environment.

## Introduction

Artificial sweeteners are widely added to foods, drinks, personal care products and pharmaceutical formulations replacing sugar in low calories diet. One artificial sweetener that has gained popularity and has been used in over 80 countries worldwide is sucralose [1,2]. Sucralose (4-chloro-4-deoxy-α,D-galactopyranosyl-1,6-dichloro-1,6-didexoy-β,D-fructofuranoside) is a chlorinated disaccharide which originates from the non-chlorinated compound sucrose (Additional file 1: Figure S1).

After decades of use of artificial sweeteners, recent studies have documented their widespread occurrence in various environmental waters, such as wastewater, groundwater, surface water, and drinking water [2-9]. Although previous studies on sucralose have proved its safety for human consumption they suggest that sucralose it is extremely persistent, with a half-life in water of up to several years, depending on pH and temperature [10]. The compound is thermally stable and not extensively adsorbed or metabolized in humans resulting in the majority (98%) being excreted unchanged. Sucralose enter the environment mainly because of incomplete removal during wastewater (conventional primary and secondary) treatment [1] and recent publications have shown that sucralose could be a valuable tracer to monitor impact of wastewaters in the environment [9].

Due to the unintended but widespread presence in the aquatic environment and the fact that long-term health effects resulting from chronic exposure to low levels of sucralose are largely unknown [6], the presence of μgL^-1^ concentrations of the sweetener in the environment has raised concern, especially since it could affect organisms feeding behaviors [11]. More alarming is the suggestion that sucralose could interfere with plant photosynthesis by shutting down CO_2_ uptake [11]. The ecotoxicological effects of sucralose still need to be systematically examined but initial studies with *Daphnia magna* and gammarids exposed to increasing concentrations of sucralose (0–500 μg L^-1^) showed that both physiology and locomotion were influenced by exposure to sucralose suggesting that sublethal effects rather than acute toxicity may be the mechanism to consider [12]. Although other studies indicated that sucralose has low toxicity and also did not bioaccumulate significantly in aquatic organisms [13-15], its persistence combined with the increasing use of this substance demands a more detailed ecotoxicological assessment [12].

The literature contains a minimal amount of research on the degradation of sucralose. Abiotic hydrolysis of sucralose does not appear to be a dominate mechanism of degradation where less than 1% of initial sucralose was shown to degrade into two chlorinated monosaccharides (1, 6-dichloro-1,6-dideoxy-D-fructose and 4-chloro-4-deoxy-D-galactose) after a 1 year incubation in a pH 3 solution at 25°C [10]. Experiments at higher, more relevant pH (4 and 6) showed no hydrolysis [10]. The first study to examine biotic degradation of sucralose found that the compound could be degraded in soil although the specific microorganism(s) responsible for the degradation were not clearly identified [16]. Labare and Alexander found that sucralose can be mineralized in natural environments, such as lake sediments (4.4–18.8%, 96–126 days), sewage (23.2%, 123 days), and surface waters (1.1–4%, 42–132 days) to lesser extents than in soils (32.6–60.4%, 20–101 days) [17,18]. The intermediates of soil microbial degradation of sucralose proposed by Labare and Alexander [17,18] either the aldehyde or the uronic acid of sucralose, could not be detected in soil incubation experiments by Soh et al. [13]. The occurrence of such intermediates is still to be reported in environmental samples [19]. Incubation experiments with sweeteners in soils, Buerge et al. reported one of the shortest half-lives (DT50) at 9 days [20]. Previous studies showed that sucralose is not oxidized by UV light or visible light [13,21]. All these studies clearly suggest that sucralose is mostly biologically inert and degrades at a slow but highly variable rate under normal relevant environmental conditions.

Robust analytical methods for assessing sucralose’s environmental fate are crucial. To date, artificial sweeteners have been determined by HPLC with reverse phase chromatography using different buffer systems, ion pairing reagents and specific derivatization procedures [5] and by GC-MS [2]. More recently, studies employing liquid chromatography–electrospray ionization-tandem mass spectrometry (LC–ESI-MS/MS) have been published for the analysis of sucralose in water samples by direct injection (higher detection limits) or offline SPE [3-6,8,22]. Nowadays the use of online SPE has shown important improvements such as higher sensitivity, analysis of smaller sample volumes, limited sample loss, no carry-over and robust and reproducible detection, while increasing sample throughput [23-27].

Although electrospray ionization is the most applied technique in LC-mass spectrometry, APCI has been shown some advantages over ESI, especially in the ionization of thermally stable polar and non-polar compounds. The ionization process associated with APCI is one of the most efficient, being considered more energetic or less soft than ESI, where gas phase reactions leading to the loss of net charge on the analyte may generate more fragment ions relative to the parent ion [28]. In fact, for certain classes of compounds that are traditionally very difficult to ionize or tend to show low sensitivity in LC-MS/MS techniques, electron capture negative APCI has provided increased sensitivity for these ‘tough to ionize’ compounds [24,29]. APCI has been reported to be more sensitive than ESI for triazines, phenylurea herbicides and organochlorine pesticides [24,30,31]. Another advantage is that signal suppression in ESI is significantly more intense than that occurring in APCI for several compounds in different matrices [32], where ionization suppression involves mainly changes in the droplet solution properties caused by the presence of non-volatile solutes in ESI ionization [33]. To this end, we have developed and validated a fast, reliable and straight-forward analytical method based on online solid phase extraction atmospheric chemical ionization mass spectrometry (SPE)-LC–APCI/MS/MS for the analysis of sucralose in reclaimed, surface and drinking waters from South Florida, U.S.A. This method was then used to evaluate the potential of sucralose photodegradation using multiple light sources and water matrices. To our best knowledge this is the first report to assess sucralose oxidation under environmentally relevant conditions.

## Experimental

### Chemicals

Sucralose was purchased from Sigma–Aldrich (Oakville, ON, Canada). Sucralose- d6 (98% purity) was used as internal standard and was obtained from Santa Cruz Biotechnology Inc, (Santa Cruz, CA, USA). Optima LC/MS grade formic acid, acetonitrile and water were purchased from Fisher Scientific (Fairlawn, New Jersey, USA). Membrane filters (0.45 μm and 0.2 μm pore size) were purchased from Millipore (Billerica, MA). Ultrapure water (>18 MΩ cm^-1^) was generated from a Nanopure Infinity Ultrapure Water system. Stock solutions of 1 mg/mL were prepared in acetonitrile for both sucralose and sucralose d-6. All stock solutions were kept in the dark at -18°C.

### Reclaimed water

“Reclaimed water can be defined as the end product of wastewater reclamation that meets water quality requirements for biodegradable materials, suspended matter and pathogens. Different applications of reclaimed water include landscape irrigation, agricultural irrigation in both food and non-food crops, ground water recharge and recreational purposes [34]. Various steps involved in producing reclaimed water from the wastewater include microfiltration through a series of membranes (0.1 to 10 μm), reverse osmosis, treatment with hydrogen peroxide followed by photolysis with UV light [35]. To date, FIU Biscayne Bay Campus receives reclaimed water from Miami-Dade North District Waste Water Treatment Facility, which has a capacity to treat 380,000 m^3^/day of water. The capacity of the existing reuse system for FIU irrigation is 1.5 million gallons per day (MGD) for irrigating 40 acres of landscape [36,37]”.

### Sample collection sites

Reclaimed water samples (n = 56) were collected at least twice monthly at Florida International University (FIU) Biscayne Bay Campus (North Miami, Florida, USA) from January 2011 to December 2011 except February2011, where only one sample was collected per month. All samples were taken directly from the sprinkler systems after they were flushed for at least 5 minutes. Drinking/Tap water samples (n = 43) were collected from residents’ homes and shopping centers located in the Miami-Dade County area. After collection, all samples were immediately transported on ice to the laboratory, filtered through a 0.45 μm glass fiber filter and then through a 0.2 μm membrane filter to minimize any potential biodegradation. Filtered samples were stored in the dark at -18°C until analysis.

Photodegradation experiments were performed using the two most common end members for treated wastewater releases, natural canal water and seawater. Canal water was collected from Tamiami Canal at its confluence with the Miami River. Seawater was taken from the shore at Bill Baggs state park at Key Biscayne, Miami, FL. Canal and seawater properties are shown in Additional file 1: Table S1. Environmental waters used to prepare the photo degradation experimental solutions were filtered twice using a 0.2-micron 47 mm glass fiber filter to remove any particles and microorganisms and then stored in the dark at <4°C until experiment was performed, typically within a week.

#### ***Online preconcentration***

A Thermo Equan online SPE system was used for the determination of sucralose in reclaimed and drinking waters. An Accela 1000 was used as analytical HPLC pump and an Accela 600 was used as loading pump (Thermo Scientific, San Jose, CA, USA). The analytical separation was carried out using a Hypersil Gold PFP column (100 mm × 2.1 mm, 1.9 μm) while the SPE pre-concentration column was a HyperSep Retain PEP (20 mm × 3.0 mm I.D) (Thermo Scientific, San Jose, CA, USA). Instrument control and data acquisition was performed using the software Xcalibur 2.1 (Thermo Scientific, USA). An HTC-PAL autosampler (Thermo Scientific, San Jose, CA, USA) was set to perform up to 10 mL injections. The automated online SPE clean-up and pre-concentration step was performed using only 10.0 mL of untreated water samples with little sample preparation. Two six-port switching valves were used for all analysis. Drinking and reclaimed water samples were prepared by adding 10 μL of 500 ng/mL sucralose-d6 and the final volume was made up to 10.5 mL with the sample.

The online procedure consists of a divert valve on the mass spectrometer which is programmed by the data system to control the loading and elution of the two LC columns. In the Load Position, 10.0 mL of sample was injected into a 10.0 mL loop and then loaded onto a SPE column by the loading LC pump (Accela 600), followed by a wash step to remove interferences (flow rate 2 mL min^-1^). The target compounds were retained in the SPE column and the matrix that is not retained during the extraction process was directed to waste while simultaneously the analytical pump equilibrates the analytical column in the starting gradient conditions. After 5.3 min, when the valve was switched to Inject Position, the solvent flow through the HyperSep Retain PEP column is reversed, and the analytes were then backflushed onto a Hypersil Gold PFP column for separation and quantitation by APCI-MS/MS. After 10 min, the switching valve was returned to the loading position to allow the extraction column to be re-equilibrated with water (A). Valve switching events as well as the gradient program are summarized in Additional file 2 for both drinking and surface waters. The samples were kept at 10°C in the autosampler. The total run time per sample was 12 min.

#### ***Direct injection LC-MS/MS method***

A direct injection method was used for the analysis of photodegradation samples. Separation was performed on a Hypersil Gold PFP column (100 mm × 2.1 mm, 1.9 μm) in 11 minutes with a flow rate of 250 μL/min using a binary gradient mobile phase consisting of acetonitrile (B) and 0.1% formic acid (C) in water according to the following program: gradient from 10% to 90% C in 6.0 min and held it for 2 min, and gradient back to 10% C in one min and held the gradient for 2 min. Column effluent was diverted to waste for the first 2.2 minutes in order to flush out the salt from the samples.

#### ***MS/MS detection***

In both methods analytes were detected on a TSQ Quantum Access QqQ Mass spectrometer equipped with an Atmospheric Pressure Chemical Ionization (APCI) source (Thermo Scientific, San Jose, CA, USA) operated in the negative mode. Preliminary tests on sucralose standards through direct injection into the LC/MS/MS were performed using HESI and APCI sources. The APCI source showed better sensitivity than HESI, moreover ESI and/or HESI spectrum generally contains abundant and extensive series of adducts ions with methanol and inorganic ions [24,38]. The APCI source was then selected and carefully optimized to produce reproducible spectra of the target compound. The optimized MS parameters were obtained by direct infusion of 10 μg/mL of standard solutions through a syringe pump at a flow rate of 50 μL min^-1^. The standard solution was mixed with the mobile phase using a T-connector before being introduced into the APCI source. The APCI vaporizer temperature and capillary temperature were 350 and 300°C respectively, with a discharge current of 5 kV. Sheath gas and auxiliary gas (N_2_) were used at a flow rate of 35 and 10 arbitrary units, respectively, and collision gas (Ar) pressure of 1.5 mTorr. The two transitions monitored for sucralose were 397 → 361 (CE, 13, for quantitation) and 397 → 359 (collision energy CE, 12, for confirmation) and for sucralose-d6 were 403→ 367 (CE, 12, for quantitation) and 403 → 365 (CE, 15, for confirmation). Instrument control and data acquisition was performed using Xcalibur 2.1 software (Thermo Scientific, San Jose, CA, USA).

#### ***UV photolysis***

Photodegradation experiments were conducted using Rayonet UV photochemical reactors (Southern New England Ultraviolet Co., Branford, CT) capable of irradiating samples at two different wavelengths (254 nm and 350 nm). Black light phosphor bulbs in the Rayonet UV reactor give out a spectrum from 320 nm to 380 nm, with a maximum at 365 nm. This band is comparable to the range of UVA region (315–400 nm) of sunlight and hence commonly used to predict the photodegradation of pharmaceutical compounds in solutions and in the environment [39]. UV 254 nm generated by mercury vapor lamps is the most widely used light source to induce photolysis of a wide variety of organic compounds and commonly used as advanced water treatment process in domestic wastewater and drinking water treatment plants. The experimental solution of sucralose was prepared by diluting the stock solution to 0.2 μg/mL with three types of water: reverse osmosis-deionized water (RODW), natural freshwater from the Tamiami canal (CW) and seawater from Key Biscayne (SW). Three 30 ml- quartz tubes were used for the kinetic experiments. The first tube filled with 30 ml of RODW was used as blank. The control tube contained sucralose but was totally covered with aluminum foil to prevent light exposure. The third tube was left uncovered and exposed to light. All the tubes were placed on a merry-go-round to ensure uniform irradiation in the Rayonet UV photochemical reactor chamber. At specified time intervals, 380 μL of the samples were transferred to a 2 mL LC amber vial, and fortified with sucralose- d6 internal standard (20 μL of 10 μg/mL) to a final volume of 400 μL. Samples were thoroughly mixed and analyzed directly by LC-MS/MS. Degradation curves assuming first order decay were plotted as [ln (C_t_/C_0_)] versus time (hrs) using the Sigma Plot software v 11.0.

#### ***SunTest photodegradation***

Sunlight plays an important role in determining the persistence and environmental fate of organic contaminants. However the intensity of natural sunlight depends mainly on the weather [40]. The intensity measured on sunny day differs from that on cloudy day and it also changes with latitude. The variable intensity of sunlight makes the kinetic parameters to vary constantly and greatly affects experimental reproducibility and interpretation. Hence experiments were conducted on a SunTest device, which is a surrogate of real sunlight. The spectra of natural solar spectrum and the SunTest are compared in Additional file 1: Figure S2. The wavelength distribution and the intensity of the Xenon lamp are very similar to those of natural sunlight [41]. Compared with the spectrum of real sunlight measured on a sunny day at Miami, FL, the light distribution of SunTest xenon lamp matched sunlight very well, especially in the 300 nm-400 nm range.

A SunTest XLS Tabletop Xenon Exposure System (ATLAS Material Testing Technology LLC, Chicago, Illinois, USA) was used to predict the degradation rates of sucralose in natural sunlight under environmental conditions. The SunTest XLS produces continuum of wavelengths from 300 nm to 800 nm by using a properly fitted Xenon lamp on the top of the exposure chamber (Additional file 1: Figure S2). The xenon lamp was used with its maximum abundance intensity (750 W/cm^2^). Test solutions of sucralose (0.2 μg/mL) were prepared in three different water matrices i.e. RODW, CW and SW. In this part of the study, 25 mL of the experimental solutions were placed in three polyethylene bags (*Nasco* WHIRL-PAK 2 OZ.), which were previously tested to be UV transparent. Similarly to UV photolysis experiments 1 control bag was kept in the dark by covering it with aluminum foil (dark control) and one was left uncovered. One bag filled with RODW acts as blank. Bags were then floated in a water bath to keep the solution at a constant temperature (25°C) when exposed under the Xenon lamp in the SunTest. The sampling interval and analytical procedures were identical as those for the Rayonet reactor experiment.

## Results and discussion

### Method development

The initial online SPE LC-APCI-MS/MS developed for determination of sucralose produced MDLs of 2.7 μg/L for reclaimed waters and 0.7 μg/L for drinking waters using 1 mL sample volume. These MDLs are adequate for wastewaters but not for surface and drinking waters. Therefore, the method was further optimized for detection of sucralose in the ppt levels (ng/L) normally observed in U.S. drinking waters [6].

**Figure 1:**
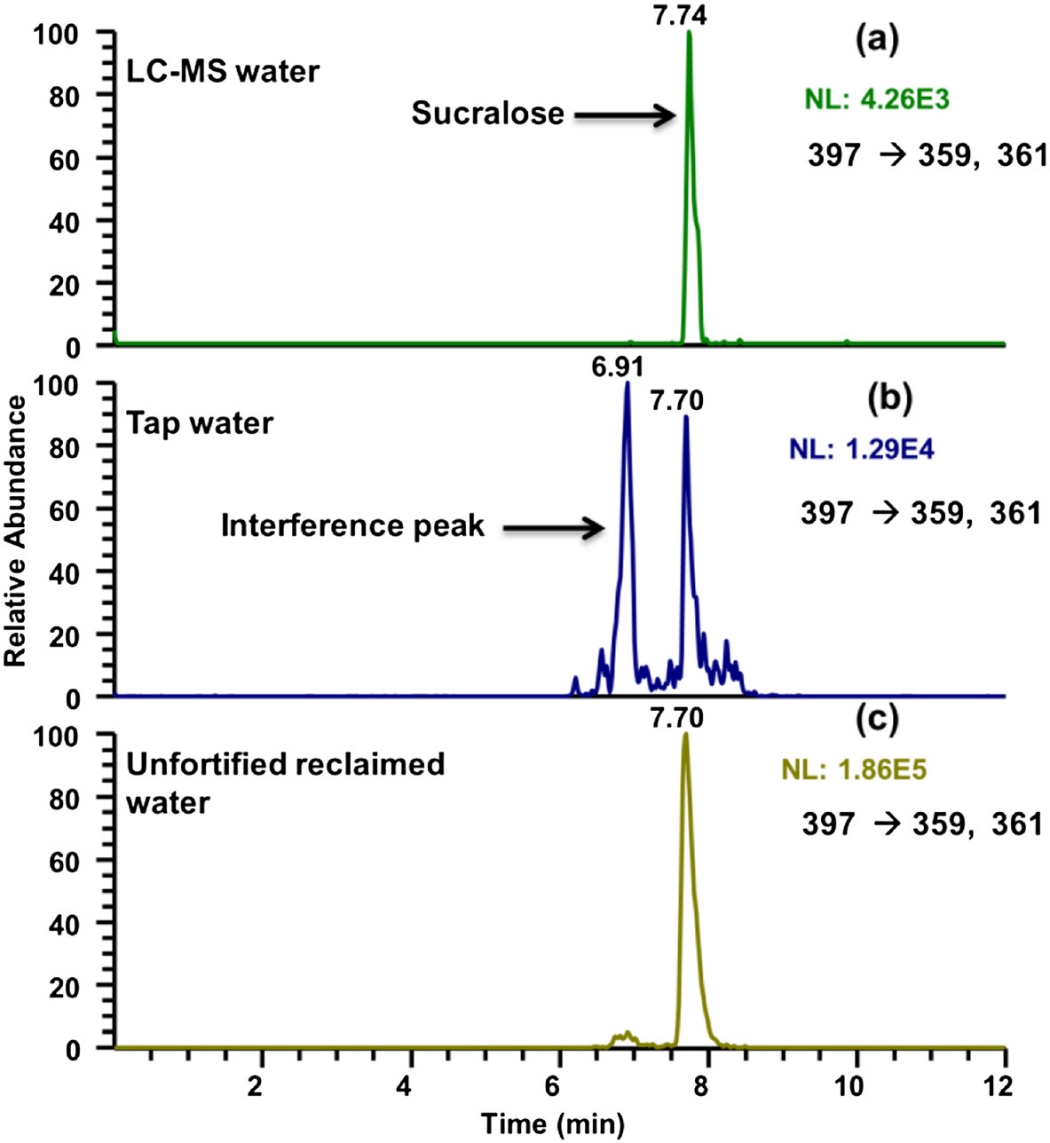
**LC-MS/MS chromatograms of sucralose in LC-MS, tap and reclaimed water. a)** LC-MS water fortified with sucralose at 100 ng/L **b)** unfortified tap water with a positive detection of sucralose **c)** unfortified reclaimed water with a positive detection of sucralose.

During the optimization process it was observed that the combination of water and formic acid was causing adverse ion suppression in the negative mode. This effect was also observed for several other compounds such as Endosulfan [24,42]. Therefore formic acid was eliminated and the mobile phase was switched to acetonitrile and water resulting in a 20-fold increase in sensitivity. Moreover, the sample volume was increased to 10 mL. The method was then able to detect sucralose at ng/L levels for all matrices. This is the first method to achieve these levels without the use of any modifier. When applying the methodology for tap water and reclaimed water samples, it was observed a high co-eluting interference with the analyte, which was not seen when formic acid was used (Additional file 1: Figure S3). In LC-MS water, no interferences were observed (Figure 1a). Separation of the interference peak from the analyte was performed by the use of a differential flow rate (Figure 1b, c). Typical chromatograms of sucralose in LC-MS water, tap water and reclaimed water can be seen in Figure 1.

### Method performance- online SPE-LC-MS/MS

The online SPE LC/MS/MS method for waters samples was validated in terms of specificity, linearity, limit of detection, matrix recoveries and inter-day precision of the technique.

The chlorine-isotopic pattern for a molecule containing three chlorine atoms such as sucralose results in four spectral peaks that differ in mass by 2 Da. The specific chlorine-isotopic pattern of the sucralose molecule was observed (m/z 395, 397, 399 and 401). The most abundant ion that was capable of giving a product ion spectrum was chosen (m/z 397). Two SRM transitions were monitored for accurate identification and quantification of sucralose.

An 8 point calibration was prepared by spiking varying levels of sucralose working standard solution in LC/MS grade water in the concentration range of 10 ng/L to 2000 ng/L. Calibration curves were built with the relative response ratio (area of sucralose divided by area of sucralose-d6) as a function of the analyte concentration. Linear response was observed in all cases (R^2^ > 0.99).

Method detection limit (MDL) was calculated from the standard deviation of seven spiked reclaimed and drinking water samples. Standard deviation of seven replicates was multiplied by the student t value at the 99% confidence interval (six degrees of freedom, t value, 3.143), according to procedures outlined by the US-EPA [43]. The matrix was spiked (n = 7) at 50 ng/L, 100 ng/L and 200 ng/L with the resulting MDLs of 4.5 ng/L, 8.5 ng/L and 45 ng/L in deionized water, drinking and 1:10 diluted reclaimed waters, respectively. Using this method, the MDL for the determination of sucralose in reclaimed waters improved by 6-fold. Matrix matched recoveries (n = 5) were assessed by spiking drinking and reclaimed and water at 50 ng/L and 200 ng/L, respectively. Because real sample matrices may contain target analytes, non-spiked samples were also analyzed and the concentration found was subtracted from the spiked sample concentration. Recoveries ranged from 91 to 108% (96.7 ± 5.27), 85 to 107% (96.2 ± 10.9) and 85 to 113 (97 ± 9.7) in deionized water, drinking and reclaimed waters, respectively.

For a repeatability study of the LC-MS method, duplicates and replicate determinations of spiked standard mixture were carried out on the same day (intra-day analysis) and on different days (inter-day analysis). The calculated relative standard deviation (RSD) ranged from 3 to 13% and 9 to 12% for intra-days and the inter-days in drinking water and reclaimed water, respectively.

Previous studies using offline sample preparation in surface water, drinking water, groundwater and sewage effluents reported MDLs in the range of 10 ng/L to 25 μg/L [3-6,8,9,22], which are consistent with the present study. Heeb et al. and Neset et al. have previously reported an online SPE LC-MS/MS determination of sucralose in wastewater, surface and drinking water; however presenting higher detection limits [26] or similar detection with larger volume of sample (20 mL) [25].

### Applicability of the method to environmental samples

**Figure 2:**
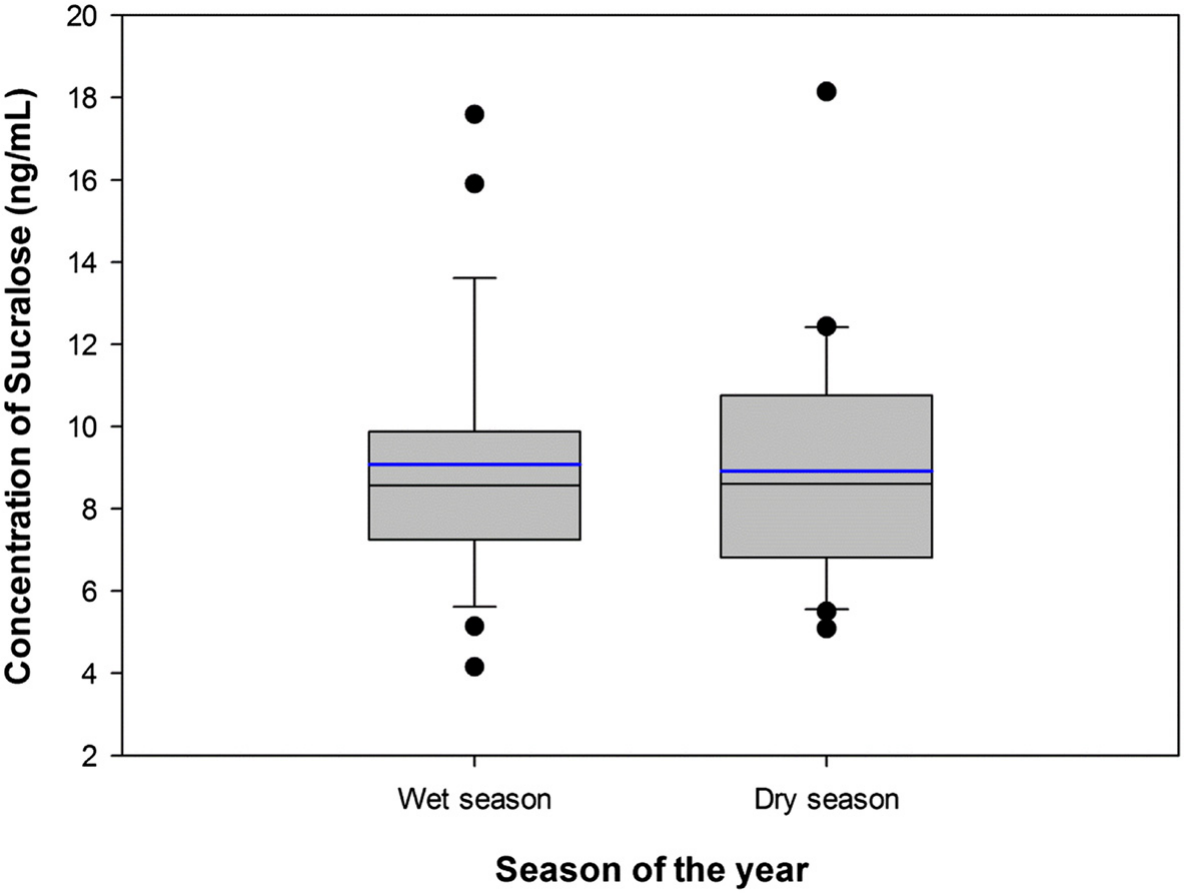
**Distribution of sucralose in reclaimed waters in various seasons.** The boundaries of box plot cover 25^th^-75^th^ percentile, the center line indicates median of the sample population, error bars (whiskers) above and below the box refer to 90^th^ and 10^th^ percentiles. The blue line in each box plot indicates mean of the sample population.

The developed online-SPE-LC-MS/MS methods were applied for the analysis of reclaimed water (n = 56) and drinking water samples (n = 43). Concentrations below MDL were considered as not detected for the purpose of average calculation. Average concentrations and frequency of detection of sucralose in reclaimed water are presented in Table 1. Sucralose was detected in 82% of the reclaimed water samples with concentrations ranging from 4.1 μg/L to 18 μg/L. The monthly average concentration was 9.1 ± 2.9 μg/L. The concentrations found in reclaimed water are comparable with previously published levels of sucralose in wastewater [8,22] and at least 10 times higher than concentrations found in surface and groundwaters [3-6,8,9]. Based on the results obtained, month wise distribution of sucralose in reclaimed water was uniform; with no observed temporal trend and statistically difference between the wet season (April to October) and the dry season (November to March) as seen in Figure 2. This result indicates that conventional Wastewater Treatment Plants (WWTP) are not efficient in removing sucralose. In fact, previous study by Brorstrom-Lunden et al. reported removal efficiency <10% for sucralose in corresponding wastewater samples [1]. The chlorinated structure of the sucralose seems to be resistant to microbial degradation even in the mixed media of a sewage treatment facility, which explains its persistence through wastewater treatment processes in municipal plants [13,21]. High consumption of sucralose in recent years along with its high persistence might explain the high concentrations observed in reclaimed water. Mass loadings (mg/day) of sucralose was calculated by multiplying its average concentration and daily flow rate of the STP effluent during the sampling period (380 000 m^3^/day). Based on the serving population of Miami Dade North District [44] the mass load per capita of sucralose was 4.37 mg/person/day, which is in good agreement with the U. S. average.

**Table 1:** Average concentration and frequency of detection of sucralose in reclaimed water samples

**Month**	**Samples/month**	**Frequency of detection (%)**	**Monthly averages (μg/L)**
Jan-11	5	80	8.00 ± 3.44
Feb-11	1	100	10.19
Mar-11	8	88	9.63 ± 4.05
Apr-11	4	100	6.96 ± 1.18
May-11	8	100	9.32 ± 2.85
Jun-11	9	89	8.53 ± 2.02
Jul-11	6	33	5.89 ± 2.47
Aug-11	4	75	10.65 ± 6.35
Sep-11	2	50	12.08 ± 1.98
Oct-11	2	100	9.42 ± 1.16
Nov-11	4	100	10.09 ± 1.34
Dec-11	3	67	8.92 ± 2.75

There is a growing concern related to the artificial sweetener sucralose in the United States and Canada. Sucralose has become the most detected unregulated chemical and artificial sweetener in wastewater, surface water and groundwater samples [19].

A worldwide comparison of the occurrence of sucralose in STPs and wastewaters is presented in Table 2 and the mass load per capita in the different countries were estimated [1,4,8,9,20-22,25,45-49]. The average effluent daily flow for China and European Union (EU) was calculated based on the general guideline suggested by Imhoff (1985), which estimates that 200 L of waste is produced per capita [26,50,51]. The estimated mass load per capita (mg/person/day) was high for U.S.A (5.0), moderate for the EU (2.1) and very low for China (0.37). The higher value observed for U.S. can be explained based on higher consumption of sucralose and its early introduction into market (1998) compared to other countries (Switzerland: 2005; Sweden: 2004; Germany: 2005; China: 2009) [19]. The mass load per capita on a global scale (2.1) was calculated similarly and is comparable to EU and lower than US.

**Table 2:** Comparison of studies on sucralose detection in sewage effluents and wastewaters

**References**	**Average concentration detected (ng/L)**	**Country**
Bronstrom-Lunden et al. 2008	3500	Sweden
Bronstrom-Lunden et al. 2008	4900	Sweden
Neset et al. 2010	2400	Sweden
Buerge et al. 2009	4470	Switzerland
Scheurer et al. 2009	800	Germany
Torres et al. 2011	2800	USA
Ordonez et al. 2012	49600	Spain
Oppenheimer et al. 2011	27000	USA
Scheurer et al. 2011	18000	Germany
Morlock et al. 2011	6449	Germany
Minten et al. 2010	11000	Sweden
Berset and Ochsenbein 2012	3641	Switzerland
Gan et al. 2013	1850	China
Current study	9100	USA
Average of all studies	10394	

**Figure 3:**
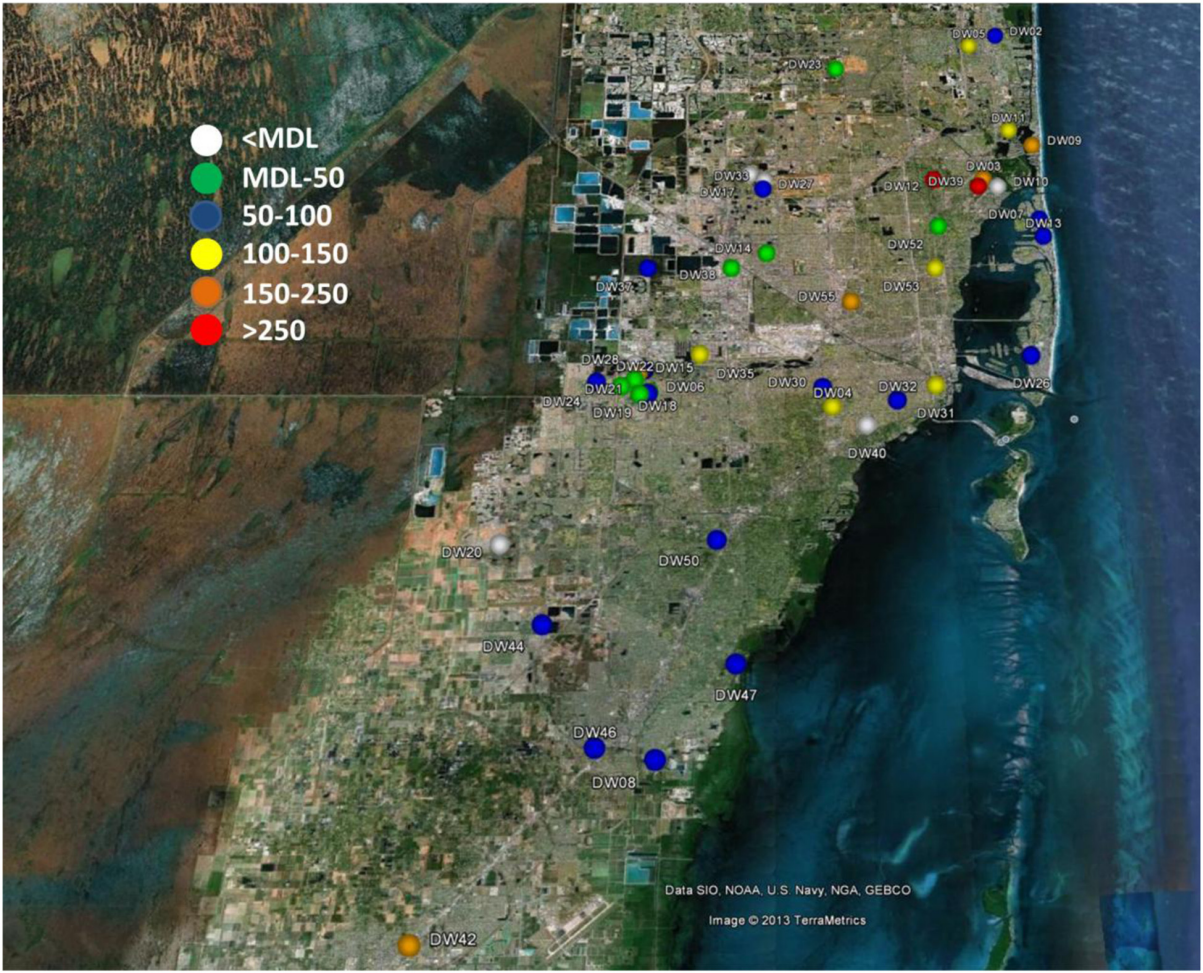
Distribution of sucralose in Miami-Dade County drinking waters.

The drinking water samples were collected from areas served by three major drinking water treatment plants in Miami-Dade, Hialeah and John E. Preston plant (n = 24), Alexander Orr Jr. plant (n = 14) and South Dade water supply system (n = 5). The Hialeah and John E. Preston plant serves most Miami-Dade residents living between the Miami-Dade-Broward County line and SW 8^th^ Street. The Alexander Orr, Jr. water treatment plant, serves most County residents living between SW 8^th^ Street and SW 264^th^ Street. The other drinking water treatment plant is South Dade Water Supply System, which is comprised of five smaller water treatment plants that serve residents south of SW 264^th^ Street in the unincorporated areas of the County [52]. The most commonly used treatment processes include filtration, flocculation and sedimentation, and disinfection. Some treatment plants also use ion exchange and adsorption. However, the exact treatment process employed in each drinking water treatment plant is not readily available. The high concentrations of sucralose in reclaimed water could be an indication that filtration by activated charcoal is not employed by these treatment plants; since previous literature showed that sucralose was effectively removed by granular activated carbon [53]. Spatial distribution of sucralose concentration in various drinking water sample locations are shown in Figure 3, with different colors ranging from white (<MDL) to red (>250 ng/L). Sucralose was frequently detected (88%) in drinking water samples with an average concentration of 111 ± 95 ng/L. The highest concentration observed in drinking water was 465 ng/L. No specific trend was observed between the concentration of sucralose in drinking waters and sampling location. Furthermore, means of Hialeah and John E. Preston plant samples and The Alexander Orr, Jr. plant samples were compared using a *t*-test and results indicated that the means of the two drinking water treatment plants were not statistically different (P = 0.142). Thus the source of variation in the concentration of sucralose among samples might be caused by the time of sample collection, residence time of the waters before distribution in drinking water treatment plant and most likely be by point sources of contamination.

Levels of sucralose in drinking water samples were similar to surface and groundwater in Europe [3,4,6,8]. Recent reports on U.S. ground and drinking waters [5,6] showed concentration of sucralose in the μg/L range, relatively higher than those reported in Europe and in the present study. For results obtained in U.S. waters, the concentrations reported here in drinking water samples (up to 465 ng/L) were lower than in surface water (up to 10,000 ng/L), drinking water (up to 2400 ng/L) and groundwater (up to 2400 ng/L) from previous studies [2,5,6,9]. These results suggest that levels of sucralose found in drinking and ground waters are comparable to surface water, demonstrating that this compound can be of great concern even for Drinking water Treatment Plants (DWTPs) with groundwater sources.

The occurrence of sucralose in groundwater and therefore drinking water would likely be an effect of surface water, contaminated with a nearby WWTP, being drawn into alluvial wells [5]. Therefore, it is reasonable to expect that human exposure to sucralose through tap water consumption may be widespread in the U.S.

### Photodegradation study of sucralose

**Figure 4:**
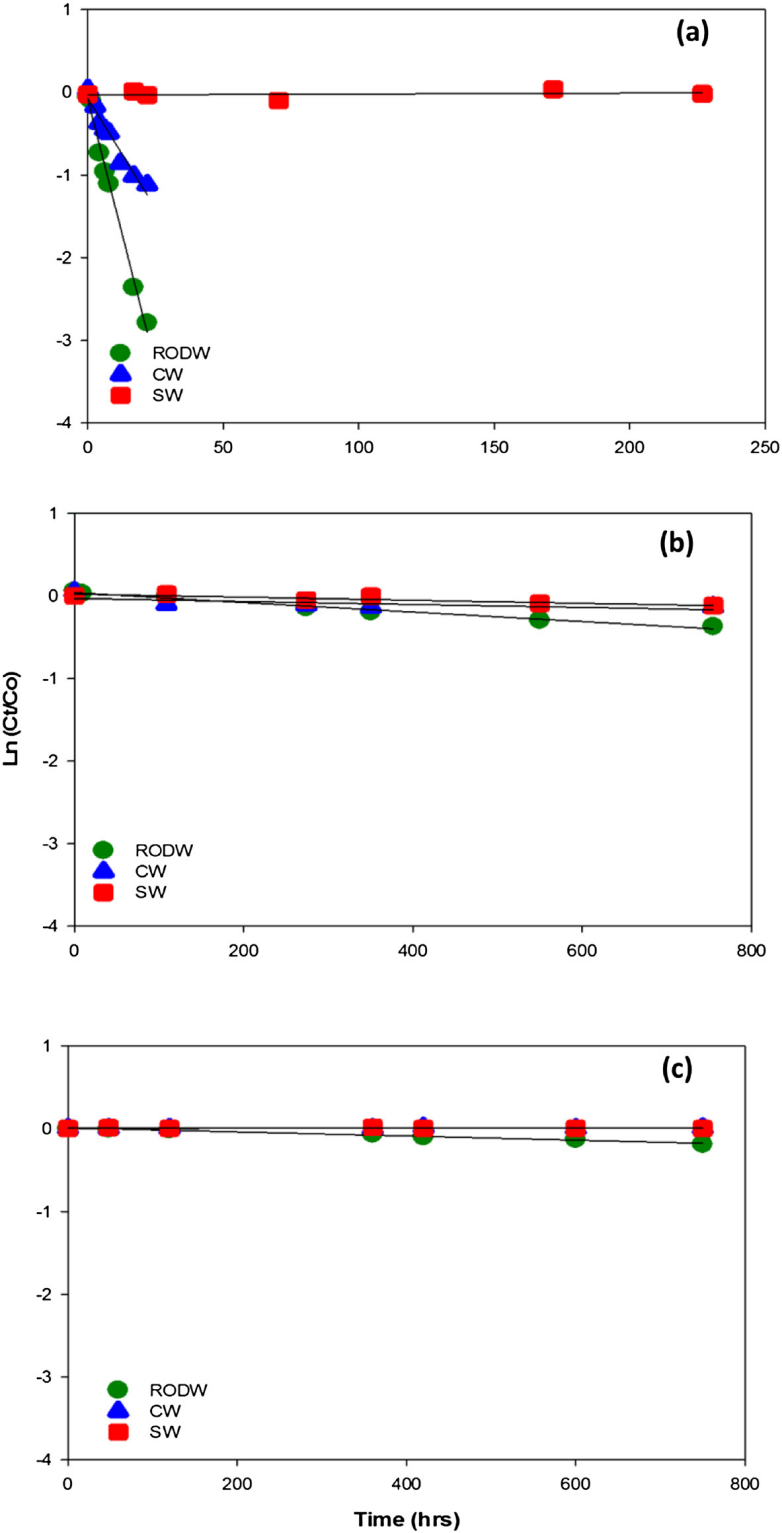
**Kinetic profile of sucralose in various light sources. a)** UV 254 nm light source **b)** UV 350 nm light source **c)** Sun Test.

The photolysis decay curves of sucralose at UV 254 nm, UV 350 nm and SunTest are shown in Figure 4 and the kinetic parameters obtained are shown in Table 3. Results clearly indicate that in all light sources, the extent of sucralose degradation is mainly dependent on the type of matrix used i.e. highest rate in RODW and lowest in salt water. In the most energetic light source used in the study (UV 254 nm) no degradation was seen in SW (Figure 4a). Similar results were observed by Torres et al. [21] i.e. minimal degradation (<8%) of sucralose in phosphate buffer was seen within 24 hours of exposure under UV 254 nm. Pronounced stability of sucralose was also evident in the UV treatment of wastewaters [21], where no sucralose degraded even after 24 hrs at 254 nm. At UV 350 nm and in Sun Test (Intensity: 750 W/cm^2^), sucralose was persistent to photolysis in both natural water matrices (SW and CW). Soh et al. reported that no degradation was seen for 1 μM of sucralose exposed to UV 254 and 320–380 nm for 5 hours [13]. After one month of continuous irradiation only <16% degraded indicating that sucralose will be extremely persistent under natural conditions (Figure 4b, c). Predicted kinetic parameters are shown in Table 3 and were calculated based on the regression equation obtained from kinetic plots.

**Table 3:** Kinetic parameters of photodegradation experiments

**Light source**	**Matrix**	**k (h**^**-1**^**)**	**r**^**2**^	**t**_**1/2 **_**(h)**	**% degradation after a month**
UV 254 nm	RODW	0.1290	0.991	5.37	100
UV 254 nm	CW	0.0530	0.948	13.1	100
UV 254 nm	SW	0.0001	0.053	>200	0
UV 350 nm	RODW	0.0006	0.984	1155	33
UV 350 nm	CW	0.0002	0.464	>750	16
UV 350 nm	SW	0.0002	0.818	>750	12
SunTest	RODW	0.0003	0.979	2310	18
SunTest	CW	0.0000	0.218	NA	0
SunTest	SW	0.0000	0.021	NA	0

### Identification of photolysis products

Kinetic photo degradation experiments showed that sucralose can be significantly degraded (t_1/2_ = 5.3 hrs for RODW) when irradiated at 254 nm. Because of that, those conditions were used to identify major degradation products. Identification of photodegradation products was carried out using high resolution mass spectrometry. A 5 μg/mL solution of sucralose was prepared in two different water matrices, RODW and SW and the spiked solutions along with blank and dark control were irradiated under 254 nm for one week and were analyzed by LC-MS/MS. Separation of the analytes was carried out on the same analytical column (Hypersil Gold PFP column) using a binary gradient mobile phase consisting of acetonitrile (B) and 0.1% formic acid (C) in water according to the following program: 100% C for the first min, gradient from 0% to 10% B in 2.0 min, then to 90% in 7.0 min, held it for 1 min, and returned back to 100% C in one min and held the gradient for 2 min. Samples were analyzed by direct injection of 20 μL of the irradiated solutions.

**Figure 5:**
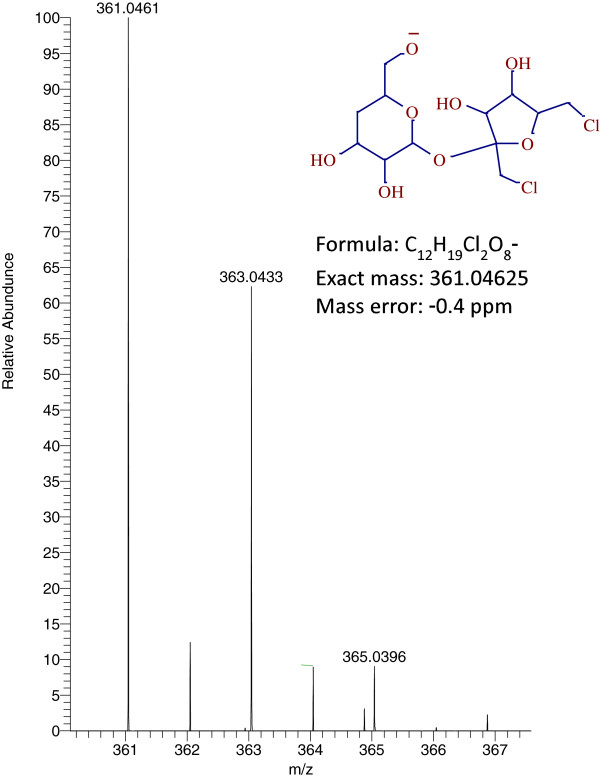
Chlorine isotopic pattern of sucralose photolysis product detected in negative ionization mode.

Quantitation was performed on a QExactive mass spectrometer (Thermo Scientific, San Jose, CA, USA) equipped with a APCI source operating in the negative ionization mode, using the following parameters: sheath gas flow: 35 arbitrary units; auxiliary gas flow: 10 arbitrary units; discharge voltage: 5 v; capillary temperature: 300°C; S-lens RF level: 90; vaporizer temperature: 350°C. Accurate mass spectra were recorded in Full scan in the range 100 to 500 m/z at a resolution of 70,000. The calculation of exact masses from elemental compositions was carried out using ChemDraw ultra 8.0. High resolution extracted ion chromatograms of the sucralose and its potential photo transformation products were obtained by processing the full scan data using Metworks 1.3 software (Thermo Scientific, San Jose, CA) with a 5 ppm mass tolerance. A signal, increasing with irradiation dose, was found at 4.4 min (0.7 min earlier than the parent molecule), with a m/z 361.0463 (C_12_H_19_Cl_2_O_8_-,-0.4 ppm) corresponding to the dechlorinated sucralose. HRMS spectra of this peak showed characteristic chlorine isotopic pattern for 2 chlorines i.e. m/z 361.0463, 363.0433 and 365.0396 with 100, 63 and 10% relative abundance, respectively as seen in Figure 5. Similar to sucralose, the photolysis product also produced a formate adduct (m/z 407.0518) with similar isotopic pattern as the parent molecule. A possible structure for sucralose degradation product (by the loss of chlorine from the six membered ring) was proposed (not shown here) based on the full scan HRMS.

Targeted MS/MS experiments were performed on both sucralose and its photolysis product at 35,000 resolution using normalized collision energy (% NCE) ranging from 10–100. When operated in negative ion mode, even at the lowest NCE used (10%), excessive fragmentation was produced, making it impossible to obtain any structural information on the photodegradation product. Alternately, Full scan and MS/MS experiments were performed using in-source collision induced dissociation (CID), which will fragment all ions after the first quadrupole. The sample was run in the full scan mode (m/z 100–500) with 3 different CIDs i.e. 0, 15 and 25 eV while monitoring the decrease of the parent ion (m/z 395.0072) and the increase of the photo-product (m/z 361.0463) and their formate adduct ions intensity (m/z 441.0130 and 407.0518). When the CID was increased from 0 to 15 eV, the abundance of both formate adduct ions decreased while the intensity of m/z 395.0072 and m/z 361.0463 increased. However, when the CID was ramped to 25 eV, the intensity of all formed ions decreased.

**Figure 6:**
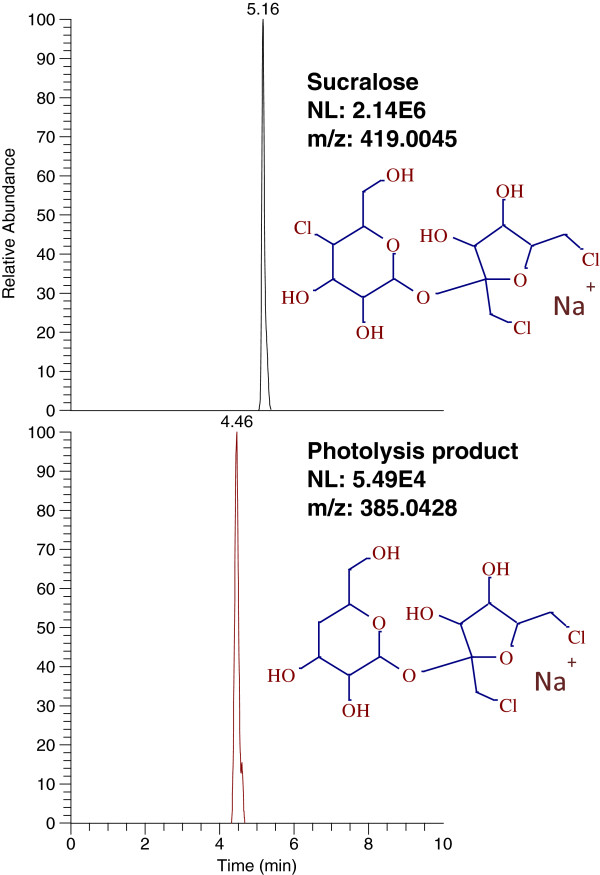
LC-MS chromatograms of sucralose and its photolysis product in positive ionization mode.

**Figure 7:**
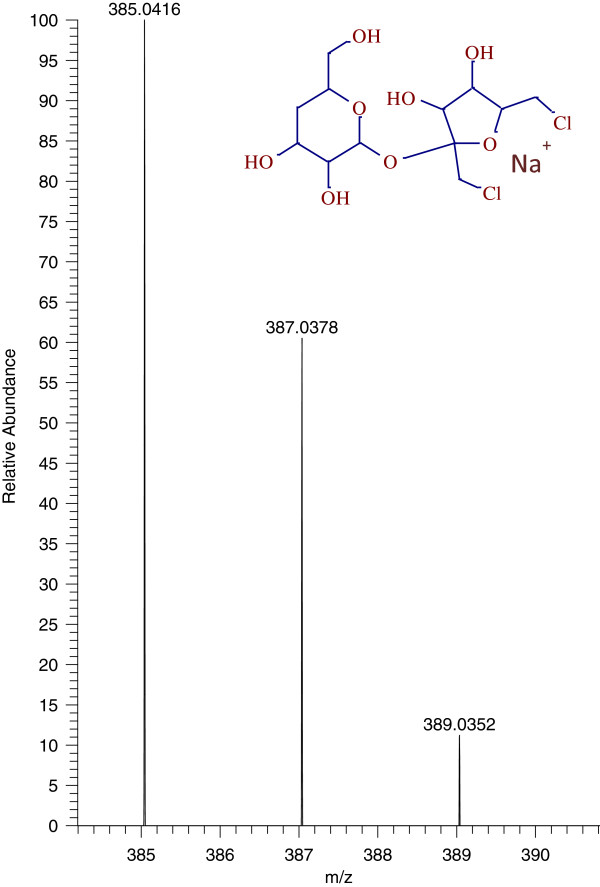
Chlorine isotopic pattern of sucralose photolysis product detected in positive ionization mode.

The same samples were then run in the positive ionization (PI) mode with same source and mobile phase conditions. Sucralose was detected in PI mode as the sodium adduct at m/z 419.0045, which is in good agreement with the results shown by Ferrer et al. 2010 [5]. However, the sensitivity in PI mode was 10–20 fold lower than in negative mode. The photolysis product identified at 4.4 min in negative mode was also identified in PI mode at the same retention time, but as a sodiated molecule at m/z 385.0428. This structure is produced by the loss of one chlorine from sucralose and corresponds to the molecular formula C_12_H_20_Cl_2_NaO_8_^+^ (+0.25 ppm). The chromatograms of sucralose and its photolysis product in PI mode are shown in Figure 6. The HRMS spectrum of the photolysis product obtained at 35,000 resolution displayed the characteristic chlorine isotopic pattern consistent with the parent molecule and is shown in Figure 7.

**Figure 8:**
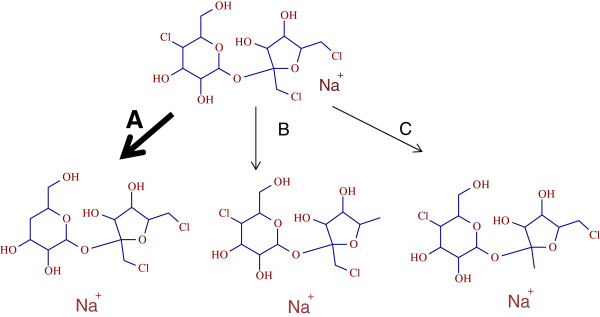
Possible structures for the sucralose photolysis product.

MS/MS information on the photolysis product was obtained at 20% NCE with an isolation window of 1 m/z and is used to elucidate the possible structure out of 3 possible ones (A, B or C) (Figure 8).

**Figure 9:**
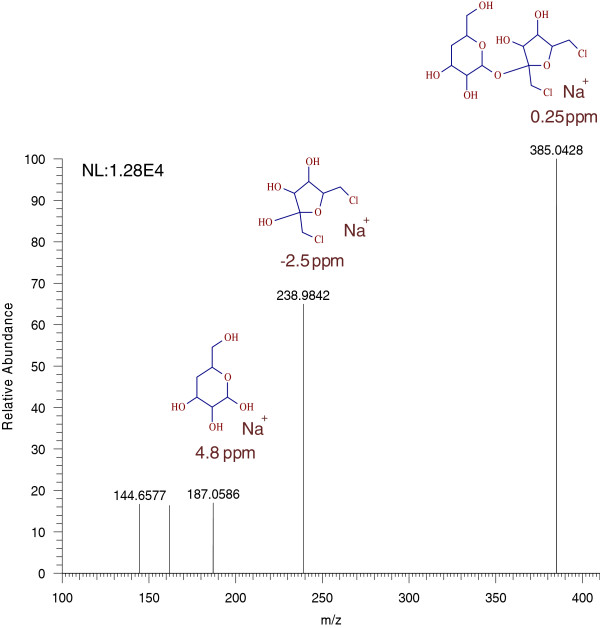
LC-MS/MS HRMS spectrum of sodiated photolysis product, m/z 385.0428.

The major MS/MS fragment at m/z 238.9842 with relative abundance of 70%, indicates that the Structure A is the most possible photodegradation product of sucralose. The MS/MS spectrum of the photolysis product, m/z 385.0428 with estimated mass errors on parent and fragments is presented in Figure 9. Chlorine isotopic pattern on MS/MS fragments was not observed due to the small isolation window selected on the parent ion (m/z 385.0428). Reclaimed water with positive detection of sucralose was run using the same method and the photo transformation product was not detected, suggesting that dechlorination of sucralose is not a preferred metabolic reaction and will occur only under prolonged UV irradiation.

## Conclusion

An automated online SPE LC-APCI/MS/MS was developed and validated for the determination of sucralose at low ng/L levels in water samples. The method was successfully applied to drinking and reclaimed waters from South Florida, U.S.A. The method detection limits were 8.5 ng/L and 2.7 μg/L in drinking and reclaimed waters, respectively. In all matrices tested, the recovery of sucralose ranged from 85-113%. Sucralose was frequently detected (> 80%) in all studied samples with concentrations as high as 18 μg/L. The mass load per capita of sucralose released by the WWTP effluent by taking into consideration all the studies published to date was estimated as 5.0 mg/day/person in the U.S. and is two times higher than the global and European Union. The maximum concentration of sucralose detected in drinking waters was 465 ng/L. Based on a study conducted by Fujimaru et al. it has been estimated that 79.5 mg of sucralose is required to make 1 L of water weakly sweet [54]. Thus, considering the calculated global mass load of sucralose discharged by the WWTPs it would still take 8.43.E + 13 years to make the ocean waters become sweet. The ubiquitousness of sucralose in the aquatic environment is however of great concern, especially since little information is known to date about its potential long term ecological effects. Photodegradation of sucralose was minimal at environmental relevant conditions. Its high resistance to photodegradation, minimal sorption and high solubility could explain the high frequency of detection and levels found in this and other studies. These results corroborate with previous findings indicating that sucralose could be a good tracer of anthropogenic wastewater intrusions into the environment. The new photolysis product identified in this study is likely produced by the loss of a chlorine directly from sucralose. To our best knowledge this is the first time that a photodegradation product of sucralose was identified.

## Competing interests

The authors declare that they have no competing interests.

## Authors’ contributions

BSR developed the online SPE-LC-MS/MS method, analyzed reclaimed waters, performed photodegradation experiments and identified photolysis products in high resolution mass spectrometry. NSQ developed direct injection LC-MS/MS method, collaborated with PVR in optimizing the method for the analysis of sucralose in drinking waters. PVR collected both reclaimed and drinking waters and trained BSR to use metabolic profiling software (Metworks). BSR, NSQ and PVR prepared and revised the manuscript. PRG procured funding, supervised the research and revised the final manuscript for publication. All the authors read and approved the final manuscript.

## Supplementary Material

Additional file 1: Figure S1 Structure of sucralose. **Figure S2**. Comparison of emission spectrum of a group of 254 nm light source, 350 nm light source and Sun Test versus natural sun light. **Figure S3**. LC-MS water fortified with sucralose at 200 ng/L (top) LC-MS water fortified with sucralose-d6 (internal standard) at 50 μg/L (bottom). 0.1% formic acid in LC-MS grade water was used as modifier. **Table S1**. Characteristics of canal water and sea water used in the experiment. Click here for file

Additional file 2: Table S2 Gradient program for sucralose determination in drinking and reclaimed water. Top left: loading pump gradient for drinking waters, top right: loading pump gradient for reclaimed waters, bottom left: analytical pump gradient for drinking waters, bottom right: analytical pump gradient for reclaimed waters. Click here for file
