# Supplementary material for: Online solid phase extraction liquid chromatography tandem mass spectrometry (SPE-LC-MS/MS) method for the determination of sucralose in reclaimed and drinking waters and its photo degradation in natural waters from South Florida
Source: Chem Cent J. 2013 Aug 22;7:141. doi: 10.1186/1752-153X-7-141 (PMC3844442; doi:10.1186/1752-153X-7-141)
Supplement: Additional file 1: Figure S1 — Structure of sucralose. Figure S2. Comparison of emission spectrum of a group of 254 nm light source, 350 nm light source and Sun Test versus natural sun light. Figure S3. LC-MS water fortified with sucralose at 200 ng/L (top) LC-MS water fortified with sucralose-d6 (internal standard) at 50 μg/L (bottom). 0.1% formic acid in LC-MS grade water was used as modifier. Table S1. Characteristics of canal water and sea water used in the experiment. [file 1752-153X-7-141-S1.docx]

**Online solid phase extraction liquid chromatography tandem mass spectrometry (SPE-LC-MS/MS) method for the determination of Sucralose in reclaimed and drinking waters and its photo degradation in natural waters from South Florida**

**Sudha Rani Batchu^1^, Natalia Quinete^1,2^, Venkata R Panditi^1,2^, Piero R Gardinali*^1,2^**

^1^Department of Chemistry and Biochemistry, Florida International University, Miami, Florida. USA and ^2^Southeast Environmental Research Center (SERC), Florida International University, Miami, Florida. USA

Email addresses:

Sudha Rani Batchu: [sbatc001@fiu.edu](mailto:sbatc001@fiu.edu)

Natalia Quinete: [nsoaresq@fiu.edu](mailto:nsoaresq@fiu.edu)

Venkata Reddy Panditi: [pvenkatreddy06@gmail.com](mailto:pvenkatreddy06@gmail.com)

Piero R Gardinali: [gardinal@fiu.edu](mailto:gardinal@fiu.edu)

*Corresponding author.

Postal Address:

3000 NE 151st ST. FIU Biscayne Bay Campus. MSB-350. North Miami Beach, Florida. 33181. USA.

Tel.: +1 305 348 6354; Fax: +1 305 348 3772


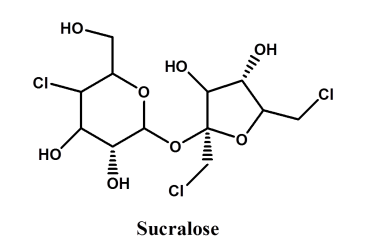


**Fig. S1** Structure of sucralose

**Fig. S2** Comparison of emission spectrum of a group of 254 nm light source, 350 nm light source and Sun Test versus natural sun light


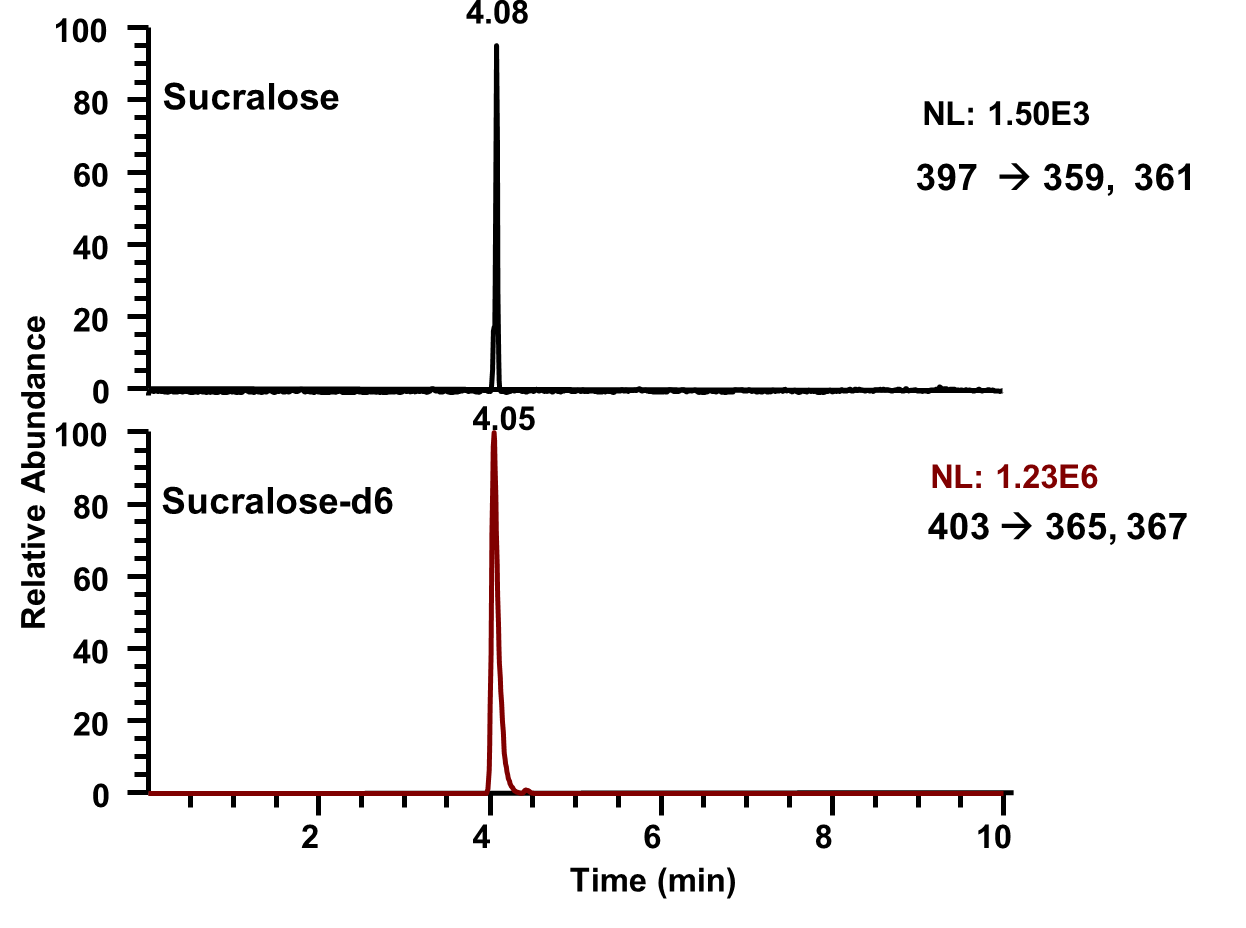


**Fig. S3**. LC-MS water fortified with sucralose at 200 ng/L (top) LC-MS water fortified with sucralose-d6 (internal standard) at 50 µg/L (bottom). 0.1% formic acid in LC-MS grade water was used as modifier.

Table S1. Characteristics of canal water and sea water used in the experiment

| Parameter | Canal water | Sea Water |
| --- | --- | --- |
| pH | 8.1 | 7.9 |
| Dissolved organic content (mg-C/L) | 10.4 | 1.37 |
| Electrical conductivity (μS/cm) | 544 | 88000 |
| Fe^+3^ (µg/L) | 187 | 109 |
| Salinity (ppt) | 0.2 | 36 |
