# Supplementary material for: Online solid phase extraction liquid chromatography tandem mass spectrometry (SPE-LC-MS/MS) method for the determination of sucralose in reclaimed and drinking waters and its photo degradation in natural waters from South Florida
Source: Chem Cent J. 2013 Aug 22;7:141. doi: 10.1186/1752-153X-7-141 (PMC3844442; doi:10.1186/1752-153X-7-141)
Supplement: Additional file 2: Table S2 — Gradient program for sucralose determination in drinking and reclaimed water. Top left: loading pump gradient for drinking waters, top right: loading pump gradient for reclaimed waters, bottom left: analytical pump gradient for drinking waters, bottom right: analytical pump gradient for reclaimed waters. [file 1752-153X-7-141-S2.docx]

Table S2. Gradient program for sucralose determination in drinking and reclaimed water. Top left: loading pump gradient for drinking waters, top right: loading pump gradient for reclaimed waters, bottom left: analytical pump gradient for drinking waters, bottom right: analytical pump gradient for reclaimed waters

| Time (min) | %A | %B | Flow  ( µL min-1) |
| --- | --- | --- | --- |
| 0.0 | 100 | 0 | 2000 |
| 5 | 100 | 0 | 2000 |
| 5.3 | 100 | 0 | 1000 |
| 6.3 | 0 | 100 | 1000 |
| 8.3 | 0 | 100 | 1000 |
| 10.0 | 0 | 100 | 1000 |
| 11.0 | 100 | 0 | 1000 |
| 12.0 | 100 | 0 | 1000 |

| Time (min) | %A | % B | %C | Flow  ( µL min-1) |
| --- | --- | --- | --- | --- |
| 0.0 | 100 | 0 | 0 | 1000 |
| 1.2 | 100 | 0 | 0 | 1000 |
| 2.0 | 0 | 50 | 50 | 100 |
| 6.0 | 0 | 50 | 50 | 100 |
| 7.5 | 0 | 50 | 50 | 1000 |
| 8.5 | 0 | 50 | 50 | 1000 |
| 8.6 | 100 | 0 | 0 | 1000 |
| 11 | 100 | 0 | 0 | 1000 |

| Time (min) | % A | % B | Flow  (µL min^-1^) **µL/min** |
| --- | --- | --- | --- |
| 0.0 | 10 | 90 | 250 |
| 2.0 | 90 | 10 | 250 |
| 5.3 | 90 | 10 | 400 |
| 8.0 | 10 | 90 | 400 |
| 12.0 | 10 | 90 | 250 |

| Time (min) | % B | % C | Flow  (µL min^-1^) µLin |
| --- | --- | --- | --- |
| 0.0 | 10 | 90 | 250 |
| 6.0 | 90 | 10 | 250 |
| 8.0 | 90 | 10 | 250 |
| 9.0 | 10 | 90 | 250 |
| 11.0 | 10 | 90 | 250 |

^A= LC-MS water; B=acetonitrile, C=0.1% formic acid in LC-MS water^
